# A Flexible Optoelectronic Device for Continuous Cerebral Blood Flow Monitoring

**DOI:** 10.3390/bios12110944

**Published:** 2022-10-31

**Authors:** Huawei Ji, Ze Xu, Mingyu Wang, Hong Zou, Ying Chen, Jun Ai

**Affiliations:** 1 School of Mechanical Engineering, Hangzhou Dianzi University, Hangzhou 310018, China; 2 Jiaxing Key Laboratory of Flexible Electronics Based Intelligent Sensing and Advanced Manufacturing Technology, Institute of Flexible Electronics Technology of Tsinghua University, Jiaxing 314000, China

**Keywords:** functional near-infrared spectroscopy, serpentine interconnector, hemodynamics, long-term monitoring, flexible optoelectronic device

## Abstract

Human cerebral oxygenation and hemodynamics can be estimated by cerebral oxygenation parameters. Functional near-infrared spectroscopy (fNIRS) can be used to measure the hemoglobin concentration index of brain tissue noninvasively and in real time. However, limited by cumbersome equipment, high price and uncomfortable wear, conventional fNIRS monitoring systems still cannot achieve continuous and long-term monitoring. In this work, a flexible and wearable long-term monitoring system is developed featured with cost efficiency, simple preparation and light weight (only 1.6 g), which consists of a pair of light-emitting diodes (LEDs) and a photodetector (PD). Triangular serpentine interconnectors are introduced to connect the functional elements, enabling the device to be stretched in multiple directions. The device can continuously work for 7 h and be subjected to 2000 cycles of bending loading, with less than 3% change in voltage value, 1.89% and 1.9% change in LED luminous power and 0.9% change in voltage value. Furthermore, the hand-gripping and breath-holding experiments show that the system can accurately measure the changes in hemoglobin concentration in accordance with the commercial device. The flexible fNIRS system presented here not only provides a simple preparation process but also offers new ideas for daily cerebral state monitoring and prolonged clinical monitoring.

## 1. Introduction

The human brain weighs only about 2% of the body’s weight but accounts for 20% of the body’s oxygen consumption [1]. Cerebral ischemia or hypoxia is an important cause of disease aggravation and even brain death, and also a common factor affecting the prognosis of patients, especially after a severe head injury, whose occurrence probability achieves almost 90%. Thus, in clinical practice, cerebral blood flow monitoring can effectively offer brain state information for guiding treatment.

The brain can be measured in non-invasive or invasive ways. Among the invasive ways, electrocortical imaging (ECoG) is the most popular method that records electrical activity from the surface or even deep areas of the brain. Although some researchers have developed microelectrode biosensors with enhanced microscale wrinkles to improve stability, the risk of cortical tissue damage still cannot be avoided, as the electrodes are directly placed on the surface of the brain [2,3,4,5]. To avoid tissue damage, non-invasive brain monitoring instruments are accepted easily and used widely, mainly including computed tomography (CT), functional magnetic resonance imaging (fMRI), electroencephalogram (EEG) and fNIRS [6,7,8,9]. CT is an easy and quick examination, capable of presenting the anatomical relationships of different cross-sectional cranial tissue structures. But some disadvantages are still obvious, involving low spatial resolution, and display failure of lesions below 1 cm. With its high spatial resolution, fMRI can show anatomical structures and diseased tissues, exhibiting high diagnostic value for neurological diseases. However, limited by calculation factors, the data collection is very slow. EEG provides higher time resolution and faster response to monitoring electrical activity. By placing electrodes on the scalp, it can be found that the electrical signal has the same change trend with brain activity. However, the biggest problem is that the spatial resolution is limited, and as it is impacted by artifacts, it is difficult to identify signal-generating locations. Functional near-infrared spectrum (fNIRS), as a completely nondestructive in vivo optical detection technique, can effectively observe neural activity in local brain regions. fNIRS has a higher time resolution than fMRI and a higher spatial resolution than EEG, as well as the advantages of being non-invasive, requiring no injection of radiographic contrast, and being easy to wear [10,11,12]. It makes it easy to realize brain state monitoring in natural contexts [13].

Over the past few years, several research groups have developed near-infrared systems for measuring brain oxygen saturation [14,15,16] in a variety of tasks for different applications. So far, in most fNIRS systems, lasers are chosen as their source of light, and avalanche photodiodes are used as the detectors to pick up light reflected off the body. Although a laser diode (LD) has a good penetration rate and can measure changes in cerebral blood flow in deeper areas, its safety is questionable. In 2013, N.L. Everdell et al. introduced a portable wireless near-infrared spatial resolution system [17]. They used LD as the light source, which required an optical fiber bundle and a complex circuit to drive the LD. The whole system was relatively large, expensive and non-portable. Many systems use fiber-optic cables to achieve proper optical coupling to the head [18,19,20]. However, the device becomes uncomfortable and non-portable for users, due to the extra weight of the optical fibers. The bare cables make the equipment vulnerable to damage when used in a natural environment. Companies such as OBELAB and Newman Brain, have designed fully integrated and portable headwear systems that enclose all of the system’s sub-components (housing, cables and electronics) completely inside the headset, shaped like a virtual reality (VR) device. These commercial systems are less dependent on skilled experts to conduct experiments and have less sensitivity to damage, although they are still too heavy to use for long-term continuous monitoring (OBELAB Nirsit-Lite: 190 g, OBELAB NIRSIT: 550 g, NewmanBrain: 290 g [21]). To become more user-friendly, these brain imaging products are tending to be miniaturized and highly portable, with considerably fewer complex electrodes and cumbersome cable connections. In 2018, Rogers et al. [22] reported a wireless, miniaturized and mechanically flexible device for cerebral blood flow detection in pediatric care. The interface materials possessed good optical and mechanical properties, contributing to efficient optical coupling and robust bonding, for high signal quality and low motion artifacts. However, the device had a complex structure and tests of the system only involved infants, with no adults.

Here, a low-cost, lightweight and easy-to-manufacture brain oxygen monitoring device is designed and fabricated. The device consists of a pair of LEDs and a PD. Serpentine interconnectors are used to connect the LEDs and PD for ductility in multiple directions, ensuring the flexibility of the device. Laser cutting, a low-cost and efficient preparation process, is used in the fabrication process, enabling a greater range of applications. The parylene package without interference in the optical path of the functional components, guarantees the stability and longevity of the device for monitoring over time. The device enables cerebral blood flow monitoring in adults and provides a means for daily and prolonged clinical cerebral monitoring.

## 2. Materials and Methods

**Figure 1:**
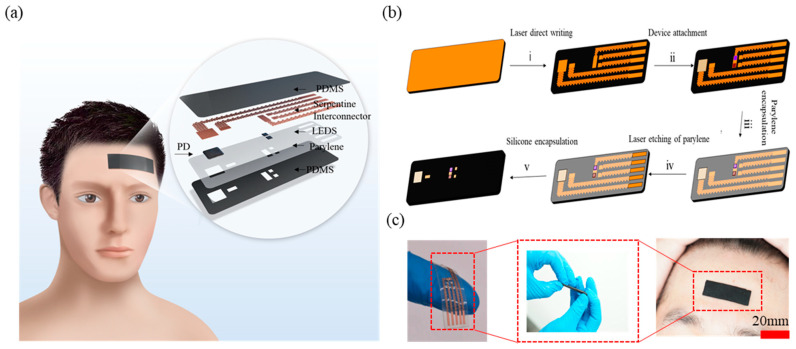
The flexible optoelectronic device. (**a**) Schematic diagram of the device for adult cerebral blood flow monitoring and the structure of fNIRS devices. (**b**) Device preparation process (patterning by laser direct writing, component bonding at low temperature, and in situ packaging). (**c**) Schematic diagram of the overall shape of the device and affixed to the forehead of a subject.

The system consists mainly of a pair of LEDs and detectors, and the selection of the right distance is the key to the system. The distance between the source and detector is generally 3–60 mm [23]. The larger the distance, the more likely it is that photons carrying deep information will reach the detector, and the deeper the detection depth, but the requirements for the light source are higher. In order to reduce the cost, LED (BN-E4242G, Epileds Technologies Inc., Suzhou, China) is selected as the light source, and the distance between the source and detector is 20 mm to detect the hemodynamics of the shallow depth of the brain. Figure 1a shows the device attached to the forehead. The device consists of transparent parylene (ZASEN NANO TECH, Suzhou, China), electronic components and a flexible interconnection layer, which is a sandwich structure, and the upper and lower layers are encapsulated by the polydimethylsiloxane (PDMS, Sylgard 184, DOWSIL, Torrance, CA, USA) mixed with black dye (smooth-on, silc-pigment, Macungie, PA, USA). The black PDMS acts as an optical barrier to eliminate ambient light interference and provides flexible packaging and protection. The intermediate layer is composed of electronic components and flexible interconnectors. The flexible interconnector is made of flexible polyimide copper-coated film (Feiyun Optical Materials, Kunshan, China) cut by laser (processing parameters: the laser average power is about 2.3 W and pulse repetition rate is 100 KHz, laser scan speed is 500 mm/s and laser scanning is repeated five times). The electronic components are bonded to the flexible circuit with conductive silver paste. The overall compact structure of the device is only 43 mm × 12 mm × 1.8 mm in size and 1.6 g in mass (Figure S1). Compared with other fNIRS systems, it has the characteristics of ultrathin and ultra-soft properties, greatly improving wear comfort [24].

Figure 1b shows the overall process of preparing the device by laser cutting. Firstly, the flexible substrate is prepared, and the liquid elastomer of PDMS and curing agent are mixed at 15:1 with a small amount of black dye. After stirring for 3 min, they are put into a vacuum drying oven (JINGHONG, Shanghai, China) and pumped for 10 min to fully exhaust the bubbles in the mixture. Then, the PDMS is poured into the mold, which is kept for about 12 h. Then the cured PDMS is peeled off to complete the preparation of the flexible substrate. The copper-coated polyimide film is manually attached to the PDMS substrate. Then, considering the feasibility, simplicity and efficiency of the process, laser cutting is used for the preparation of the interconnects. Compared to complex photolithography processes, laser cutting is mask free, and efficient due to its low cost. To ensure the flexibility of the device in multiple directions, we have designed triangular serpentine interconnectors. The triangular serpentine enables the device to stretch in several directions compared to common interconnects. The electronic components are attached to the flexible interconnect layer with conductive silver paste and their pads are bonded to the circuit by gold wire bonding. The surface of the prepared device is evaporated with a layer of parylene which insulates and protects the surface of the components and ensures that the system has a stable operation. The parylene encapsulation also prevents the gold wire from falling off easily from the pad during the device’s working process. The device is placed into the mold, through the guide hole, and into the uncured black PDMS. After the curing of the black PDMS, the preparation is finished. Figure 1c shows the appearance of the device before and after packaging. Without packaging, the device is transparent, ultra-thin and ultra-soft. After packaging, the device avoids the interference of ambient light and can be integrated with the head.

**Figure 2:**
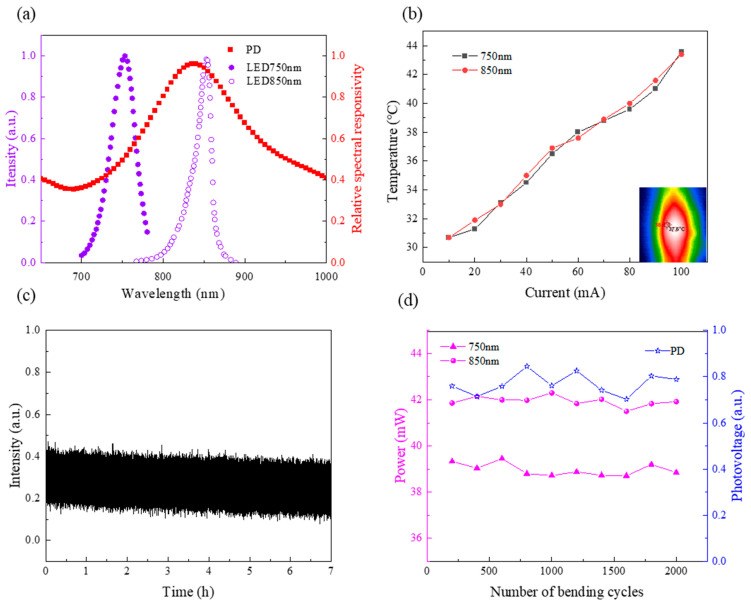
Optical, thermal and electronic characteristics. (**a**) Light emission spectrum characteristics for sensor probe LEDs and PDs. (**b**) Measurement of LED temperature as a function of current. (**c**) The flexible fNIRS device continuously monitors the voltage value of PD for 7 h. (**d**) LED luminescent power and PD response voltage after 2000 bending cycles.

In the preparation process of flexible devices, the selection of components is very important, which affects the signal-to-noise ratio of the collected data. The light source wavelength is chosen properly as the most abundant substances in human tissues are water and protein, which have a low absorption rate in the near-infrared band (650 nm–900 nm) [23]. At this time, human tissues are more transparent, and the near-infrared light can pass through the human tissues and be detected by detectors. The LEDs with wavelengths of 750 nm and 850 nm are selected as the light source in this system, which are respectively located above and below the equal absorption point (805 nm) of oxy-hemoglobin (HbO_2_) and deoxy-hemoglobin (HbR) to maximize sensitivity. The actual luminous wavelength of LEDs is generally discrete, that is to say, its actual luminous wavelength may have some error compared with the nominal value. If simply the extinction coefficient of HbO_2_ and Hb corresponding to the actual nominal value of LEDs is used, the final calculation result will have an obvious error. Figure 2a shows that the actual wavelengths of the LEDs are 752 nm and 851 nm, revealing the extinction coefficient of HbO_2_ and HbR of the two wavelengths [25]. At the same time, the responsivity of the photodetector test is also important, and it is verified to have a high response in the near-infrared band. PD has a lower power consumption, which ensures the device’s performance and security and achieves long-term monitoring. Temperature is more important for the real application of the device, mainly because the light source is constantly emitting heat, while the paste of the device limits the heat exchange between the skin and the external environment. In Figure 2b, the actual temperature of the LEDs is tested as a function of current. The temperature changes linearly with the current. At first, when the current is small, the LED light-emitting temperature is close to the ambient temperature. Then, when the current increases to 100 mA, the temperature is close to 40 ℃, which may cause discomfort to the subject. The final current is chosen, in which the surface temperature is close to the human body temperature.

The device is placed in a dark box, a constant 50 mA current is applied to the two LEDs, the sampling frequency is set to 10 Hz and the data is collected by the PD to test the stability of devices for long-term monitoring. In Figure 2c, during the 7 h of data collection, the voltage values of the PD remain stable and fluctuate by less than 3%, indicating that the device can be used for accurate monitoring over a long period. The device is fixed on the stretching machine (CARE Measurement&Control, Tianjing, China) and the number of cycles of loading the stretching machine is set to 2000; each cycle in turn is set to a period of 10 s, with an initial phase of 270° and a displacement of 8 mm to test the stability of the device after cyclic loading of the device. The luminous power of the LEDs and the voltage values collected by the PD in a dark room are recorded after each cycle with the same input current. As seen in Figure 2d, the power variation of the LEDs at both wavelengths is only 1.89% and 1.9%, and the voltage variation value of the PD is even less than 1%. Thus, the device is verified with a long lifetime and performance stability over a long time.

**Figure 3:**
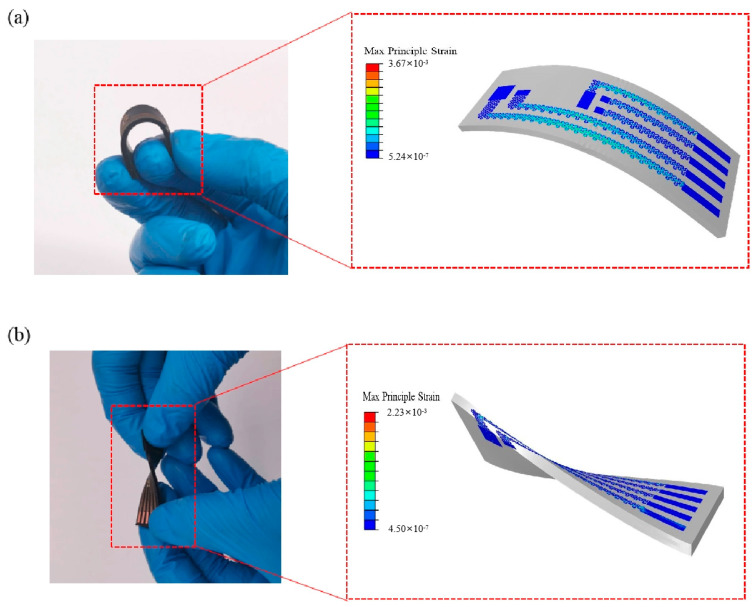
Optimized mechanics for the flexible device. (**a**) Strain distribution calculated by FEA in the copper layers of the device with a bending radius of 3 cm. (**b**) Strain distribution of the copper layer for a device with a torsion angle of 40°.

As shown in Figure 3a,b, the device can be bent and twisted, enabling a gentle and nonirritating interface with robust optical and mechanical coupling to the skin. The finite element analysis (FEA) results in Figure 3a indicate that the maximum principal strains in the interconnect layer are below 0.3% (the limit for plastic deformation) for a bending radius of 3 cm, which is much smaller than that required for a neonatal forehead. Under bending and twisting, the device performs with low strain in most areas. Figure 3b indicates that the maximum principal strains in the interconnect layer are below 0.25% for a twist angle of >40°. It proves that the device is easy and safe to be bent and twisted, and will not constrain the skin when it is affixed to the forehead, contributing to comfortable wearing.

## 3. Results and Discussion

Performance measurements and structural simulations were completed to verify that the device is capable of long-term testing of human hemodynamic parameters and adapting to a variety of environments. A series of experiments were designed to verify the effectiveness and sensitivity of the device. The sensitivity of the device was initially tested in an in vitro experiment. A forearm blocking experiment was set up to test the effectiveness of the device by applying pressure to the forearm with a sphygmomanometer bandage to block the venous return and arterial blood supply to the forearm of the subject. To validate that the device can test the changes in hemoglobin concentration in the brain, a basic experimental study was conducted on healthy adult participants. The breath-holding and hand-gripping experiments are chosen as the experimental paradigm. Using the modified Beer–Lambert law (MBLL) [26], the changes were calculated in HbO_2_ and HbR over time. The raw brain data was accompanied by heartbeat (1–1.5 Hz), breathing (0.2–0.5 Hz) and Mayer waves (−0.1 Hz), using a fourth-order Chebyshev bandpass filter with a pass band of 0.01 Hz–0.25 Hz to remove the noise from the fNIRS signal.

### 3.1. In Vitro Experiment

**Figure 4:**
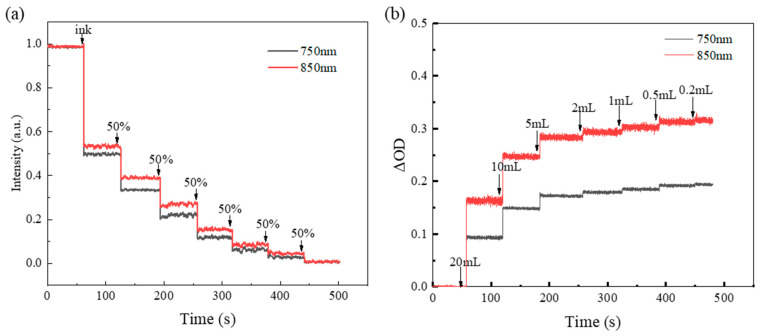
Results of in vitro experiment. (**a**) Liquid optical model experiment results for 750 and 850 nm. (**b**) Blood model experiment results for 750 and 850 nm.

To test the sensitivity of the devices, the liquid optical model experiments and blood model experiments were carried out. During the liquid optical model experiment, 600 mL water was poured into the container, diluting the carbon black ink to 50% of the previous concentration every minute, as shown in Figure S2a. Due to the continuous injection of ink, the reflection of light will become weaker and weaker, and the voltage value will also show a step-down trend. Figure 4a shows that the two wavelengths have similar shapes and corresponding steps. The liquid optical model preliminarily verifies the effectiveness of the device, the increasing liquid concentration leads to weaker light reflection and the device can detect the change in light intensity after six times liquid dilution, showing high sensitivity. During the blood model experiment, 900 mL phosphoric acid buffer (PBS) was poured into the beaker to simulate the physiological environment of human tissues [26], with 34 mL fat milk solution as the scattering medium. The fresh rabbit blood was injected into the container by turning on the magnetic stirrer (Figure S2b), and the change in optical density (ΔOD) was recorded. The blood injections were in the order of 20, 10, 5, 2, 1, 0.5 and 0.2 mL, with changes in concentration of 50%, 25%, 10%, 5%, 2.5% and 1% compared with the initial (20 mL) injected. Figure 4b shows the step increase in optical density (ΔOD). As the change in concentration becomes smaller, the step between two adjacent steps shrinks. Until the injection of 0.2 mL, the step of optical density (ΔOD) variation disappears and a relatively stable rising line is realized [24]. As seen from the experiments, the device can detect a 1% change in blood volume, demonstrating a high level of sensitivity.

### 3.2. Forearm Block Experiment

To validate the functionality of the system, arterial occlusion and isometric voluntary forearm muscle contraction were performed on healthy male subjects. Figure S3a shows the device is placed above the elbow of the anterior arm to remove the blood flow to the hand. The sphygmomanometer around the upper arm was inflated to 220 mm Hg, lasting 30 s, and then the cuff gas was released to restore blood flow. Figure S3b shows the changes in the subject’s HbO_2_ and HbR over time. After the pressure is applied, the superficial veins and deeper arteries are blocked, so the concentration of hemoglobin in the tissue remains unchanged; at the same time, the tissue metabolism consumes oxygen, so the oxyhemoglobin concentration is decreasing and the HbR is increasing. At 60 s, the sphygmomanometer starts to release pressure, in order to compensate for the reduction in oxygen to the tissues caused by the blocked blood vessels, the fresh blood flows rapidly to the arm and the concentration of HbO_2_ in the arm rises rapidly and that of HbR falls rapidly; subsequently, both hemoglobin concentrations gradually become stable. In comparison with experimental results from the literature, it is found that our device performs a similar trend [26,27], proving the validity of the device in practical human testing.

### 3.3. Prefrontal Cortex fNIRS Experiment

**Figure 5:**
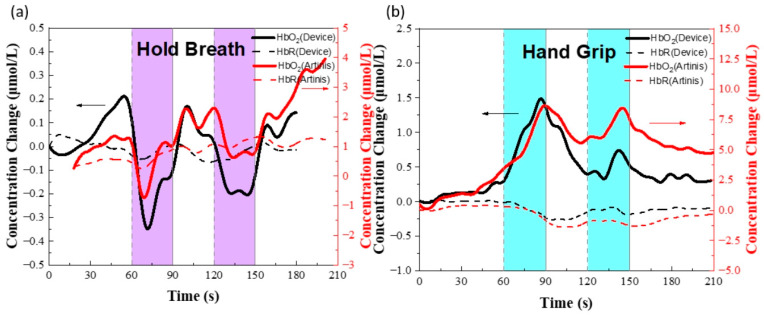
Data collection of systemic and cerebral hemodynamics from healthy young adults. (**a**) HbO_2_ and HbR measurements from our device and a commercialized fNIRS instrument (named Artinis) during a physiological validation using a breath-holding test. Complete session data. The solid black lines represent device HbO_2_, the red lines are Artinis HbO_2_, and the shaded purple areas represent breath-holding periods. Characteristic dips and recovery in HbO_2_ are visible in these data. (**b**) Our device and a commercialized fNIRS instrument validation using a hand-gripping test.

To verify that the device measures changes in hemoglobin concentration in the prefrontal lobe (hairless area) accurately, the device was attached to the prefrontal cortex of the subject’s brain. The Valsalva test is a physiological test commonly used to non-invasively evaluate the regulation of cerebral blood flow by increasing intrapleural pressure to reduce the return of blood from the veins to the heart, thereby altering the body’s normal blood circulation [28]. Healthy adult subjects were asked to complete normal breathing for 60 s, hold their breath for 30 s and repeat twice. Before each breath hold, the subject was asked to take a deep breath. The results of the relative changes in ΔHbO_2_ and ΔHbR in the prefrontal cortex are calculated from all raw intensity measurements as shown in Figure 5a. The experiment begins with the subject breathing normally and the changes in ΔHbO_2_ and ΔHbR concentrations are insignificant, fluctuating up and down by less than 0.2 μmol/L. When the subject inhales deeply to increase the internal thoracic pressure and then holds his breath, the oxyhemoglobin concentration decreases rapidly and tends to continue to rise during the breath-holding period. The amount of change in both concentrations decreases and then rises to bulge a small packet when the test subject resumes normal breathing. The whole trend of change is consistent with available physiological studies [29,30,31]. To demonstrate that the results of the device testing are not coincidental, a second set of experiments was set up, again by attaching the device to the prefrontal cortex region of the subject’s brain.

The changes in hemoglobin concentration in the prefrontal lobe were simulated by means of hand-grip, for the same duration as that of Valsalva, with a 60 s rest period and a 30 s fist clench repeated twice. The results of the experiment are shown in Figure 5b. At the beginning of the experiment, the oxyhemoglobin and deoxyhemoglobin concentrations remained stable due to the absence of external stimuli, and when the fist-shaking stimulation was performed, the oxyhemoglobin concentration rose and reached an amplitude at 25 s, followed by a continuous decrease in oxyhemoglobin concentration until the second stimulation. The oxyhemoglobin concentration gradually increased after a slight delay. The reduced hemoglobin concentration remains almost constant throughout the process. From the above results, it can be seen that our device can measure changes in hemoglobin concentration well, and the comparison of trends with commercial devices verifies the validity and accuracy of the device.

## 4. Conclusions

In this study, we developed a flexible and wearable near-infrared spectrometer device, and introduced triangular serpentine interconnectors into the device, allowing it to adapt well to the skin. The parylene packaging without interference on the optical path of the functional components guarantees the stability and longevity of the device for monitoring over time. Laser cutting, a low-cost and efficient preparation process, is used in the fabrication process, enabling a greater range of applications for health monitoring in low-resource environments. To demonstrate its stability, the device was tested for 7 h of continuous operation with less than a 3% change in the voltage value of the detector. Its sensitivity was verified using previous in vivo experiments, achieving an accuracy of 1% for the detection of blood volume increments. A series of experiments were conducted to test its accuracy in measuring the relative changes ΔHbO_2_ and ΔHbR. The results of the hand-gripping and breath-holding tests show that they can accurately measure changes in hemoglobin concentration, consistent with commercial equipment. The device proposed in this paper offers new ideas for flexible electronic technology processes and the possibility of continuous brain monitoring on a daily and long-term basis.

## Data Availability

The data presented in this study are available on request from the corresponding author. The data are not publicly available due to privacy and ethical concerns.

## Supplementary Materials

The following supporting information can be downloaded at: https://www.mdpi.com/article/10.3390/bios12110944/s1, Figure S1: Weight and Size of the device. (a) The weight of the device is recorded. (b) A vernier caliper rec-orded the thickness of the device; Figure S2: Set up in vitro experiment. (a) Set up of liquid optical model experiment. (b) Set up of blood model experiment; Figure S3: The image and results of the forearm blocking experiment. (a) Set up of the forearm blocking experiment. (b) The results of the experiment; Table S1: Cost analysis of the device in this study.
