# Supplementary material for: A Flexible Optoelectronic Device for Continuous Cerebral Blood Flow Monitoring
Source: Biosensors (Basel). 2022 Oct 31;12(11):944. doi: 10.3390/bios12110944 (PMC9688213; doi:10.3390/bios12110944)
Supplement: Supplementary file 1 [file biosensors-12-00944-s001.zip › biosensors-1987148-supplementary.pdf]

biosensors

Supporting Information

A Flexible Optoelectronic Device for Continuous Cerebral Blood Flow Monitoring

Huawei Ji [1], Ze Xu [1], Mingyu Wang [1], Hong Zou [1], Ying Chen [2] and Jun Ai [2,*]

1 School of Mechanical Engineering, Hangzhou Dianzi University, Hangzhou 310018, China
2 Jiaxing Key Laboratory of Flexible Electronics based Intelligent Sensing and Advanced Manufacturing Technology, Institute of Flexible Electronics Technology of Tsinghua University, Jiaxing, 314000, China
* Correspondence: aijun@ifet-tsinghua.org; Tel.: +86-13554327723

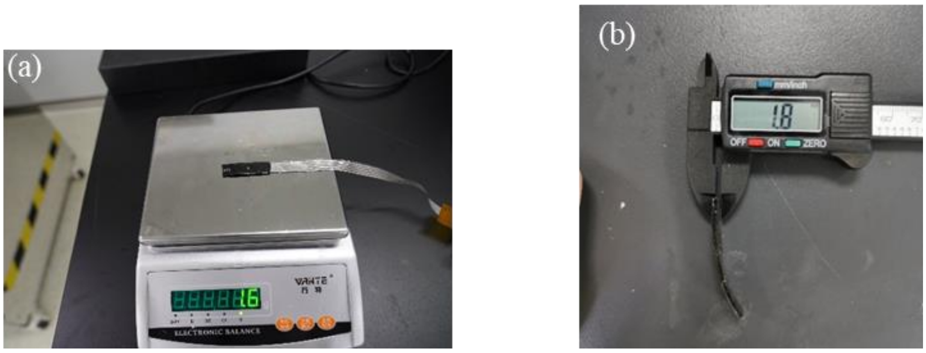

Figure S1. Weight and Size of the device. (**a**) The weight of the device is recorded. (**b**) A vernier caliper recorded the thickness of the device.

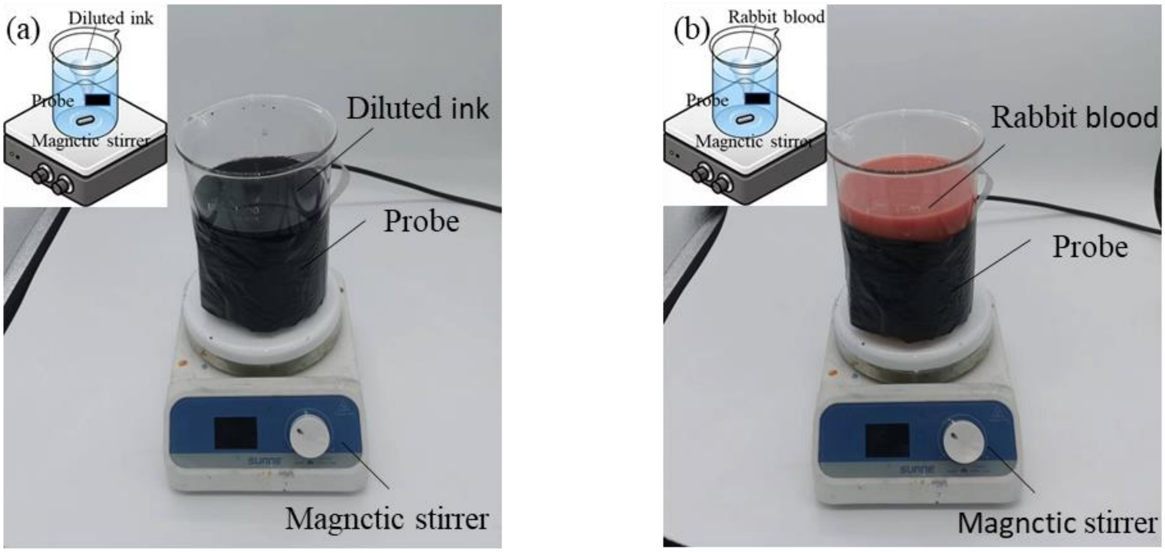

Figure S2. Set up in *vitro* experiment. (**a**) Set up of liquid optical model experiment. (**b**) Set up of blood model experiment.

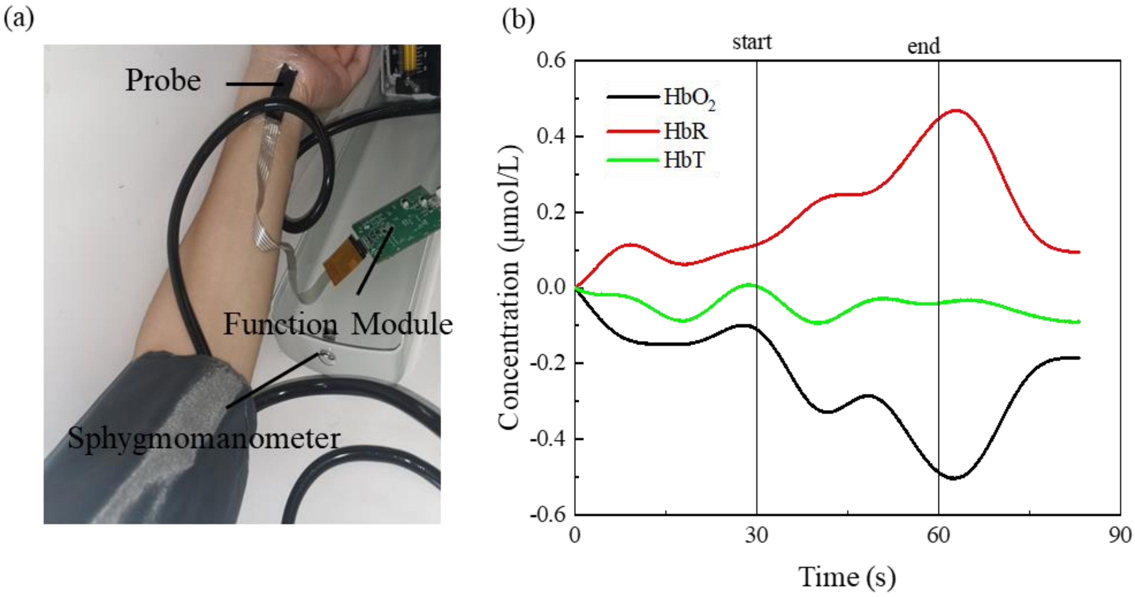

Figure S3. The image and results of the forearm blocking experiment. (**a**) Set up of the forearm blocking experiment. (**b**) The results of the experiment.

Table S1. Cost analysis of the device in this study.

Components	Cost Determination
750 nm LED & 850 nm LED	$0.5
PD	$7
Flexible Polyimide Copper	$0.5
Fabrication Cost	$0.8
PDMS & Black Dye	$1
